# Melatonin improves spatial navigation memory in male diabetic rats

**Published:** 2012

**Authors:** Farrin Babaei-Balderlou, Samad Zare

**Affiliations:** *Department of Biology, Faculty of Sciences, Urmia University, Urmia, Iran.*

**Keywords:** Diabetes, Melatonin, Spatial navigation memory, Hippocampus, Streptozotocin

## Abstract

The aim of the present study was to evaluate the effect of melatonin as an antioxidant on spatial navigation memory in male diabetic rats. Thirty-two male white Wistar rats weighing 200 ± 20 g were divided into four groups, randomly: control, melatonin, diabetic and melatonin-treated diabetic. Experimental diabetes was induced by intraperitoneal injection of 50 mg kg^-1^ streptozotocin. Melatonin was injected (10 mg kg^-1^ day^-1^, ip) for 2 weeks after 21 days of diabetes induction. At the end of administration period, the spatial navigation memory of rats was evaluated by cross-arm maze. In this study lipid peroxidation levels, glutathione-peroxidase and catalase activities were measured in hippocampus. Diabetes caused to significant decrease in alternation percent in the cross-arm maze, as a spatial memory index, compared to the control group (*p* < 0.05), whereas administration of melatonin prevented the spatial memory deficit in diabetic rats. Also melatonin injection significantly increased the spatial memory in intact animals compared to the control group (*p* < 0.05). Assessment of hippocampus homogenates indicated an increase in lipid peroxidation levels and a decrease in GSH-Px and CAT activities in the diabetic group compared to the control animals, while melatonin administration ameliorated these indices in diabetic rats. In conclusion, diabetes induction leads to debilitation of spatial navigation memory in rats, and the melatonin treatment improves the memory presumably through the reduction of oxidative stress in hippocampus of diabetic rats.

## Introduction

Diabetes mellitus is a common metabolic disorder characterized by hyperglycemia due to insulin deficiency.^1^ The long-term hyperglycemia increases glycation proteins and lipids, enhances glucose auto-oxidation, which in turn generates reactive oxygen species (ROS). These species lead to the chronic oxidative stress in diabetes mellitus.^2^^,^^3^ Oxidative stress is reported to specifically impair the memory function in experimental animals with strong involvement of the hippocampus.^4^^,^^5^ The hippocampus has maintained a central position in the development of psychological theories of normal and abnormal human and animal memory for the last 40 years or so. There is a large body of evidence showing that the activity of hippocampal neurons in both rats and primates reflects information about the spatial organization of an animal’s environment.^6^ Neuroimaging studies in humans have provided evidence that the hippocampus becomes active during spatial navigation and some researches suggest that the hippocampus in rats may contribute to more than one type of spatial navigation. Path integration is a form of navigation in which an animal integrates self-movement cues to locate its present position or to return to a starting location. Support for the role of the hippocampus in path integration comes from both electrophysiological and lesion studies in rats.^6^^- ^^8^

Since the diabetes mellitus-induced oxidative stress has an effective role in hippocampus damage^3^^,^^5^ and spatial memory impairments^9^^,^^10^ we have hypothesized that anti-oxidants could ameliorate the cognitive deficits by decreasing the amount of ROS in diabetic rats. 

Melatonin has been suggested as an antioxidant that may reduce lipid peroxidation, which is an indicator of oxidative stress in the rat brain.^11^^,^^12^ Melatonin readily passes all cell membranes, including the blood-brain barrier.^13^ It has been shown that melatonin scavenges several free radicals including the peroxyl and hydroxyl radicals. Both these radicals can initiate lipid peroxidation.^14^^,^^15^ Additionally, melatonin also increases the activity of glutathione peroxidase in the brain.^16^

There is evidence about efficacy of melatonin on learning and memory and its protective effect on hippocampus.^4^^,^^5^^,^^10^ However, to the best knowledge of the authors, the role of melatonin on spatial navigation memory has not been elucidated. Therefore, the purpose of this study was to assess the possible advantage effect of melatonin due to its antioxidant activity on spatial navigation memory in diabetic rats.

## Materials and Methods

**Animals and treatments. **Thirty-two male white Wistar rats weighing 200 ± 20 g each were used in the study. The animal room temperature was maintained at 22 ± 2 ˚C, under a 12 hr/ 12 hr light/dark cycle. Food and water were available *ad libitum*. All animals were randomly divided into two groups: control and diabetic. Animals were rendered diabetic by a single intraperitoneal injection of 50 mg kg^-1^ streptozotocin. Streptozotocin was dissolved in 0.05 M citrate buffer at pH 4.5 immediately before administration. Control rats (n=8) were injected with the vehicle alone. Blood glucose levels as a parameter of diabetes mellitus were determined using a glucometer (ACON Laboratories, Inc., USA) and a tail vein 72 hr later. The rats with hyperglycemia (glucose higher than 220 mg dL^-1^) were considered as diabetic. Maturing animals exposed to chronic hyperglycemia manifest pathological alterations in central nerve structure and function.^17^ Accordingly three weeks after streptozotocin injection, glycemia was again determined and all rats with a final blood glucose levels above 220 mg dL^-1^ and all control rats were randomly assigned to two groups (each were included eight rats): the first group of diabetic and of control rats received daily melatonin at a dose of 10 mg kg^-1^ intraperitoneally. Melatonin was dissolved in ethanol and this solution was then diluted with saline to a final volume (final concentration of ethanol, 4%). The second group of diabetic and control rats was injected with vehicle alone. All solutions were intraperitoneally injected at a volume of 0.1 mL per 100 g body weight for 12 days. All chemicals were purchased from Sigma (Sigma-Aldrich Corp., St. Louis, MO, USA). The experimental protocol was reviewed and approved by the Local Institutional Committee for the Ethical Use of Animals.

**Spatial navigation memory test. **This test was performed using cross-arm maze. This maze has been used in laboratories associated with cognition. The purpose of the test is for the rat to remember which arm was last visited and try to enter as many different arms as possible. The task is testing hippocampal navigation memory and can be weakened by lesions to the hippocampus.^18^ This model was used to test spatial navigation memory with respect to spatial orientation and perception as described by Ragozzino *et al*.^18^ Briefly, rats were removed from the cages and placed individually in a four-arm cross maze. The maze constructed of wood, painted gray and contained a central platform (25 cm diameter) from which radiated four symmetrical arms (55 cm long × 10 cm wide) with 12 cm walls. After being placed in the central platform, rats were allowed to transverse the maze freely for 12 min. The number and sequence of entries were recorded; an alternation was defined as entry into four different arms on an overlapping quadruple set. Four consecutive arm choices within the total set of arm choices made up a quadruple set. A quadruple set consisting of arm choices B, D, C, A comprised as ‘Actual alternation’ while the set with B, D, B, A did not (using this procedure, possible alternation sequences were equal to the number of total arm entries minus three). 

Percent alternation was calculated as follows:

$\mathrm{Alternation \%}=\frac{\mathrm{Actual alternation}}{{\mathrm{Possible alternation}}^{*}}\times 100$

*Number of total arm entries – 3.

Alternation percent is an indicator of spatial navigation memory of rat experimented and the number of total arm entries is the index of locomotor activity in this maze.

**Biochemical measurements.** The hippocampus were homogenized in 1:10 (w/v) cold 25 mM potassium phosphate buffer (pH=7.4) and used to determine lipid peroxidation, glutathione peroxidase and catalase activities. Malondialdehyde (MDA) levels were estimated by the method of Esterbauer and Cheeseman.^19^ The degree of lipid peroxidation was assessed according to MDA formation, which is accepted as an index of lipid peroxidation. Malondialdehyde, an end product of fatty acid peroxidation, reacts with thiobarbituric acid (TBA) to form a colored complex. The principle of the method is the spectrophotometric measurement of the color generated by the reaction of the TBA with MDA. For this purpose, 300 μL of 10% trichloroacetic acid were added to 150 μL of each sample and centrifuged at 1000× *g* for 10 min at 4 ˚C. Three hundred microliters of the supernatant were transferred to a test tube and incubated with 300 μL 0.67% thiobarbituric acid at 100 ˚C for 25 min. The mixture was allowed to cool on water for 5 min. The resulting pink stained TBA-RS were determined in a spectrophotometer at 535 nm. TBA-RS were quantified using an extinction coefficient of 1.56 × 10^5^ M^-1^ cm^-1^ and expressed as nmol of MDA per g wet tissue. Glutathione peroxidase activity was measured according to the method of Lawrence and Burk.^20^ The enzymatic reaction was initiated in the tube that contained reduced nicotinamide adenine dinucleotide phosphate, reduced glutathione, sodium azide and glutathione reductase by the addition of cumene hydroperoxide (CuOOH) and the change in absorbance at 340 nm was monitored with a spectrophotometer. Activity is given in unit per mg protein. Catalase activity was assayed measuring the absorbance decrease at 240 nm in a reaction medium containing 30 mM H_2_O_2_, 50 mM potassium phosphate buffer pH 7.0 and 50 μL of the sample, according to Aebi method.^21^ One unit of enzyme is defined as one μmol of H_2_O_2_ consumed per min and the specific activity is reported as unit per mg protein. Protein concentrations were determined according to the method of Lowry *et al*.^22^

**Statistical analysis. **All data were analyzed using one way analysis of variance (ANOVA) and group differences were determined using Tukey’s test, by SPSS version 15.0 statistical software package for Windows (SPSS Inc., Chicago, IL, USA). In all calculations a difference at *p* < 0.05 was regarded as significant.^23^ All results are expressed as mean ± SEM in table 1.

## Results

**Fig. 1:**
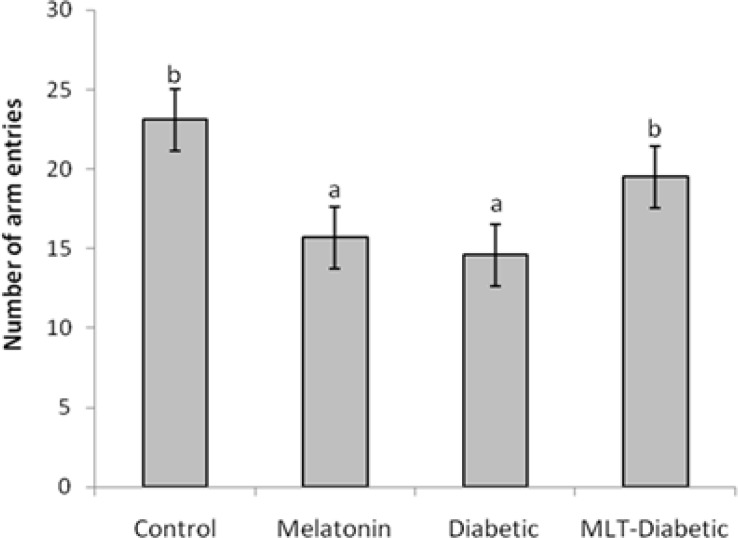
Effect of diabetes and melatonin on number of entries in the cross-arm maze in rats (Mean ± SEM). ^a^ indicates significant difference compared to control group (*p* < 0.05). ^b^ indicates significant difference compared to diabetic group (*p* < 0.05).

**Fig. 2:**
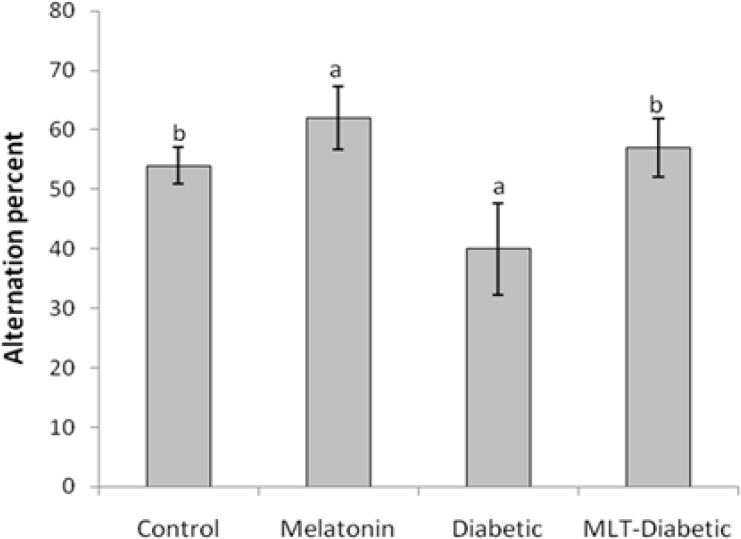
Effect of diabetes and melatonin on alternation percent in the cross-arm maze in rats (Mean ± SEM). ^a^ indicates significant difference compared to control group (*p* <0.05) ^b^ indicates significant difference compared to diabetic group* (p* <0.05).

**Effect of diabetes and melatonin on spatial navigation memory.** Figure 1 shows the number of arm entries, as a locomotor activity index, in cross-arm maze significantly (*p* < 0.05) decreased in diabetic rats as compared to the controls. Treatment of diabetic rats with melatonin caused to significant (*p* < 0.05) increase in locomotor activity compared to the diabetic group. The number of arm entries significantly (*p* < 0.05) decreased in rats injected with melatonin alone compared to the control animals. Figure 2 shows the percent alternation in cross-arm maze. Diabetes caused to significant decrease in actual alternation score accordingly alternation percent, the spatial navigation memory index, in the cross-arm maze compared to the control group (*p* < 0.05), whereas administration of melatonin significantly (*p* < 0.05) prevented the spatial memory debilitation in diabetic rats. Also melatonin injection significantly (*p* < 0.05) increased the alternation percent in intact animals compared to control animals.

**Table 1:** Effects of melatonin on the activity of antioxidative enzymes and malondialdehyde levels in hippocampus (Mean±SEM).

Parameters	Control	Melatonin	Diabetic	Melatonin-treated diabetic
Malondialdehyde nmol g^-1^ wet tissue	10.68 ± 1.02 b	8.74 ± 1.03 b	17.06 ± 1.15 a	11.23 ± 0.93 b
Glutathione peroxidase U mg^-1^ protein	0.07 ± 0.00 b	0.06 ± 0.00 b	0.05 ± 0.00 a	0.06 ± 0.00 b
Catalase U mg^-1^ protein	0.32 ± 0.03 b	0.33 ± 0.03 b	0.27 ± 0.01 a	0.32 ± 0.01 b

a indicates significant difference compared to control group* (p* < 0.05).

b indicates significant difference compared to diabetic group* (p* < 0.05).

**Effect of diabetes and melatonin on Biochemical parameters in hippocampus. **Assessment of hippocampus homogenates indicated a significant (*p* < 0.05) increase in lipid peroxidation levels and a significant (*p* < 0.05) decrease in GSH-Px and CAT activities in diabetic group compared to the control animals, while melatonin administration significantly (*p* < 0.05) ameliorated these indices in diabetic rats. No significant difference was observed between melatonin treated rats and control group (Table 1).

## Discussion

Previous studies have reported that diabetes mellitus is associated with neurological complications in both the peripheral and central nervous system.^1^^,^^3^^,^^24^ Impairment of learning and memory is also recognized as a complication of diabetes.^5^^,^^9^^,^^10^^,^^25^^,^^26^ In animal models of diabetes, such as the streptozotocin-induced diabetic rats, spatial memory impairments have also been reported.^26^^,^^27^ Cognitive deficits in diabetes mellitus can result from metabolic impairment and hyperglycemia.^2^^,^^3^ Although the pathogenesis of these deficits is multifactorial and controversial, but there is strong evidence for the involvement of oxidative stress due to excess production of reactive oxygen species (ROS).^2^^,^^3^ The mammalian hippocampus, which plays a pivotal role in a diverse set of cognitive functions such as spatial memory, is very vulnerable to oxidative damage in diabetic animals.^28^^-^^30^ In agreement with this idea, it has been reported that lipid peroxidation enhances in hippocampus, which in turn leads to a significant impairment in memory behavioral functions in diabetic animals.^5^^,^^31^^-^^33^ In the present study, administration of streptozotocin significantly increased malondialdehyde levels, an index of lipid peroxidation, in the studied hippocampus. One reason for the elevated lipid peroxidation in streptozotocin-induced diabetes is the reduction of antioxidant enzymes such as glutathione peroxidase and catalase activities.^5^ In this experiment we found that untreated diabetes caused reduced activities of glutathione peroxidase and catalase in hippocampus. Our findings are consistent with the previously published reports.^31^^-^^33^ It has been shown that the antioxidant enzymes activities were decreased in hippocampus in chronic experimental diabetic neuropathy.^5^^,^^31^^-^^33^

Different studies indicate that various ROS scavengers ameliorate cognitive deficits through the hippocampus protection.^5^^,^^33^ In the current study we have examined the effects of treatment with melatonin on the spatial navigation memory in diabetic rats. The treatment was aimed at reducing the oxidative stress in hippocampus; accordingly we investigated the levels of lipid peroxidation and the antioxidant enzymes activities in hippocampus.

In our study, treatment of diabetic animals with melatonin significantly reduced lipid peroxidation in the hippocampus. Furthermore, we found that a decrease of glutathione peroxidase and catalase activities in mentioned area of brain was reversed by the administration of melatonin. Considerable evidence identify melatonin as potent antioxidant and its protective effects against oxidative stress in different areas of brain have been reported to date.^14^^-^^16^

In this study we have further examined the effects of melatonin on spatial navigation memory in diabetic rats by cross-arm maze. We showed that diabetes could significantly lead to the decrease of actual alternation percent as a spatial navigation memory index. Despite previous studies indicate deficit in spatial learning and memory, as revealed by the Morris water maze, T-Maze and Y-maze, in streptozotocin-induced diabetes,^5^^,^^10^^,^^26^^,^^27^^,^^30^^,^^31^ it has not been demonstrated the effect of diabetes on spatial navigation memory. However, oxidative stress may contribute to spatial navigation deficit due to the hippocampus impairment during hyperglycemia. Therefore, antioxidants could be relevant options to use in the prevention of hippocampal damage associated with diabetes. Herein we found that melatonin as an antioxidant could increase the alternation percent during the cross maze test. Ragozzino *et al*. have shown the correlation between the alternation percent and the function of hippocampus by cross-arm maze.^18^ Therefore, it has been speculated that melatonin has improved the spatial navigation memory as a function of hippocampus in diabetic rats.

Despite some evidence identifying melatonin as a potent antioxidant in hippocampus,^4^^,^^5^^,^^11^ its improvement effect on spatial navigation memory in diabetic rats has not been reported to date. This is the first report to show that melatonin improves this type of memory against oxidative stress. Nevertheless, beneficial effects of melatonin on spatial learning and memory and function of hippocampus in several experimental diabetic and non-diabetic models have been reported previously.^4^^,^^5^^,^^10^^,^^34^ Tuzcu and Baydas have shown that oxidative stress may contribute to learning and memory deficits in diabetes and melatonin can improve the cognitive impairment in diabetic rats.^5^ In Rimmele *et al*.’s study, melatonin exerted a genuinely central nervous effect by enhancing encoding of objects under stress and improved memory acquisition.^34^ Nedzvetsky *et al*. have indicated that melatonin prevents the development of cognitive deficiency in diabetic animals.^10^ Despite these evidences about advantage effects of melatonin on memory Gönenç *et al*. have shown that treatment of rats with melatonin did not affect the spatial memory impairment due to acute ethanol exposure by Morris water maze testing.^4^ Recently the other study indicated while the removal of the pineal gland and exogenous administration of melatonin via injections are causing impairment, constant-release melatonin administration via implantation does not affect the learning performance and the spatial memory.^35^

The different effects of melatonin on spatial memory between some previous studies and the present study could be due to the test employed in these studies. Morris water maze, almost employed in above studies, has been reported to be sensitive to some exogenous modifications such as nutrition procedure, stress, infection, etc. as well as endogenous factors such as sex, age and strain differences. Mentioned factors were different in these experiments; as well as the cross-arm maze and the other mazes used in previous studies reflect the different types of spatial memory due to hippocampus function.

Herein we also found the inhibitory effect of melatonin on locomotor activity in non-diabetic rats. We observed that the number of arm entries, as a locomotor activity index, in cross-arm maze decreased in diabetic rats. Treatment with melatonin caused to increase in locomotor activity, as well as increased alternation percent in diabetic rats; whereas melatonin increased the alternation percent and decreased the locomotor activity in non-diabetic rats. This contradiction indicates that increasing effect of melatonin on actual alternation score could not be a result from the locomotor activity increscent in diabetics. We speculated that melatonin probably exerts different mechanisms in managing the spatial memory under the either oxidative stress or normal conditions in rats. 

In conclusion, this study indicated the role of melatonin in the protection of spatial navigation memory in streptozotocin-induced diabetic rats probably through the enhancing antioxidant status of the hippocampus. 
